# To Drain or not to Drain? Point-of-care Ultrasound to Investigate an Axillary Mass: Case Report

**DOI:** 10.5811/cpcem.2022.2.53357

**Published:** 2022-04-18

**Authors:** Kishan Patel, Zara Khan, John Costumbrado

**Affiliations:** * University of California, Riverside School of Medicine, Riverside, California; † Riverside Community Hospital/University of California, Riverside, Department of Emergency Medicine, Riverside, California

**Keywords:** point-of-care ultrasound, POCUS, abscess, lymph node, case report

## Abstract

**Introduction:**

Point-of-care ultrasound (POCUS) has great sensitivity in the diagnosis of abscesses and swollen lymph nodes. Many studies outline the characteristics that distinguish abscesses from lymph nodes on POCUS.

**Case Report:**

We present a case from the emergency department in which a patient presented with a potential abscess but was found to have a malignant lymph node on imaging.

**Conclusion:**

Point-of-care ultrasound can be used to differentiate an abscess from a swollen lymph node. Abscesses are generally anechoic or hypoechoic with septae, sediment or gas contents, and they lack internal vascularity. Benign lymph nodes are echogenic with hypoechoic cortex with hilar vascularity.

## INTRODUCTION

Evaluation of abscesses versus enlarged lymph nodes is an important distinction to be made in the emergency department (ED) prior to performing incision and drainage in a patient who presents with a “lump.” Point-of-care ultrasound (POCUS) is a diagnostic tool that has been shown to have a greater sensitivity than physical exam alone to diagnose both abscesses and enlarged lymph nodes in the ED.^1^,^2^,^3^,^4^,^5^ Previous ED studies have shown that POCUS can allow clinicians to diagnose abscesses with significant accuracy and speed, often changing medical management as a result.^6^,^7^ Previous studies have also characterized distinctive qualities of abscesses and lymph nodes that can be used to confirm the presence of one as opposed to the other.^4^,^5^,^7^,^8^,^9^,^10^,^11^ From experiences in the ED with patient presentations requiring accurate identification of an abscess against a lymph node, we recognized that POCUS can play an instrumental role in the rapid detection and distinction between the two.

The echogenicity of abscess on sonography varies based on the characteristics of the abscess itself. However, a common distinguishing factor for all abscesses is the absence of internal vascularity.^3^,^12^ Lymph nodes, on the other hand, typically display significant hilar vascularity, with malignant nodes having mixed or absent hilar vascularization.^4^,^8^ However, unlike the varied echogenicity of an abscess, lymph nodes are frequently small, oval, and hypoechoic in appearance.^4^,^5^ With an understanding of the common characteristics and qualities of both abscesses and lymph nodes, benign or metastatic, we believe POCUS can be used in conjunction with a thorough physical exam to guide medical management. We present a case in the ED where a patient presented with signs and symptoms of an abscess but instead was confirmed to have an enlarged lymph node, raising suspicion for underlying metastatic disease.

## CASE REPORT

A 54-year-old female with a past medical history of kidney transplantation, hypertension, and mitral valve replacement presented to the ED for two days with a painful lump in her left axilla. She was seen at urgent care two days prior and prescribed oral clindamycin. The lump did not change in size after the antibiotic; she denied fevers or discharge from the area. She had a prior history of a tooth abscess. A 10-point review of systems was otherwise negative. The patient stated she wished to have the “abscess” drained.

Her vitals were unremarkable. Her physical exam was significant for a a firm lump in the left center axilla visible on inspection and palpation, three centimeters (cm) in diameter and at the site of the expected central axillary nodes. No erythema or active drainage was noted. Her laboratory findings were significant only for a normocytic anemia with hemoglobin of 9.6 grams per deciliter (g/dL) (reference range: 12.0–15.5 g/dL).

**Image 1:**
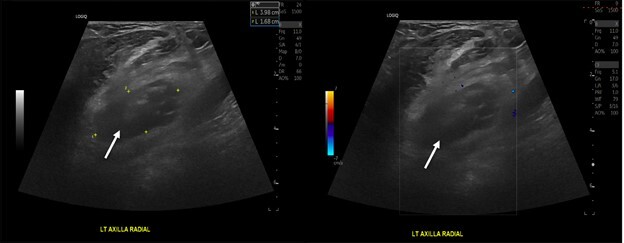
(Left panel) White arrow indicates point-of-care ultrasound (POCUS) image of abnormal left axillary lymph node on B-mode. (Right panel) White arrow points to POCUS image of abnormal left axillary lymph node with no vascularity on color Doppler mode.

Despite the patient’s expressed concern that she had an abscess and that she was being treated with outpatient antibiotics, and requested that the suspected abscess be drained, we performed a point-of-care ultrasound (POCUS) because the physical exam findings did not obviously indicate an abscess. The POCUS findings were more consistent with a lymph node prompting further imaging studies, and no incision and drainage was performed (Image 1).

Computed tomography (CT) chest without intravenous (IV) contrast revealed an enlarged, left supraclavicular lymph node measuring 2.5 x 4.6 cm. In addition, a 1.5 x 2.4 x 2.3 cm soft tissue nodule with surrounding edema representing an enlarged lymph node with superimposed infection was found in the left axilla. We were unable to obtain a contrast study as the patient was refusing IV access at the time after discussing risks, benefits, and alternatives. Overall, the findings were suspicious for nodal metastases. A breast ultrasound found left breast hypoechoic mass at the two o’clock position, two cm from the nipple and 3.9 cm in size, along with an enlarged left axillary lymph node.

**CPC-EM Capsule**

What do we already know about this clinical entity?

*Point-of-care-ultrasound (POCUS) is used widely in emergency medicine to diagnose abscesses and lymph nodes*.

What makes this presentation of disease reportable?

*This report discusses a patient with a suspected abscess requesting drainage; however, POCUS results suggested an alternative diagnosis, which altered management*.

What is the major learning point?

*Point-of-care-ultrasound can be used to differentiate an abscess from a lymph node using specific sonographic features*.

How might this improve emergency medicine practice?

*Understanding the sonographic differences can allow immediate identification and influence management of lymph nodes or abscess in the emergency department*.

## DISCUSSION

There is growing interest in using POCUS to identify abscess and lymph nodes in the ED. In this case, we present a report of a patient with a suspected abscess who was found to have evidence of possible metastatic disease on CT. Hence, it is crucial to identify the sonographic differences between an abscess and an enlarged lymph node as it can influence management.

**Image 2:**
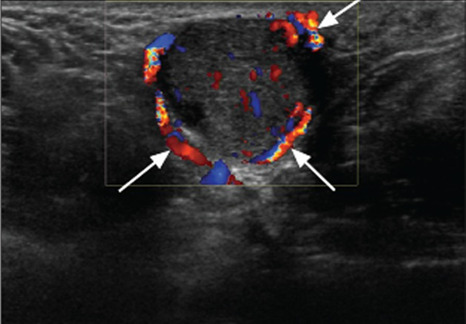
White arrows indicate point-of-care-ultrasound color Doppler image of axillary abscess showing minimal central vascularity, increased peripheral vascularity, posterior acoustic enhancement, and round hypoechoic central contents of abscess.^14^

Early ultrasound studies have shown that an abscess possesses several unique sonographic patterns.^3^,^11^ A well-formed abscess has been shown to appear as round, anechoic, or hypoechoic masses with posterior enhancement and septae, sediment, or gas contents.^11^,^13^ Most are also surrounded by some cellulitis or edema leading to a cobblestoning appearance near the fluid collection. Further, ultrasonic fluctuance is common where gentle pressure over the abscess leads to abscess contents swirling.^11^,^13^ Color Doppler often reveals a lack of internal vascularity – a common distinguishing characteristic of an abscess (Image 2).^3^,^14^

**Image 3:**
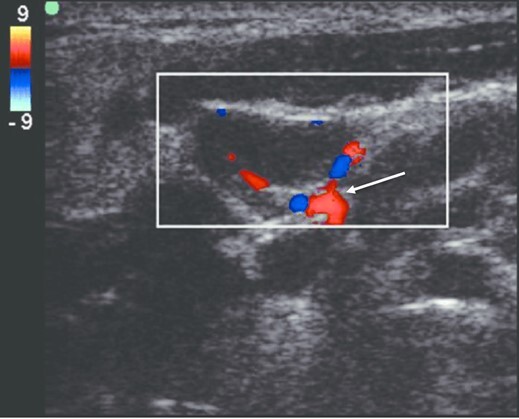
White arrow points to point-of-care-ultrasound color Doppler image of benign cervical lymph node showing hilar/central vascularity.^15^

Ultrasound elastography has also been found to distinguish the induration of an abscess from surrounding healthy tissue.^13^ With these characteristics, POCUS was found to have about 98% sensitivity with 69–88% specificity, much greater than a physical exam sensitivity of 78% and specificity of 66%.^1^,^2^ Studies have also looked at using sonographic patterns to identify benign and metastatic lymph nodes.^4^,^5^,^8^ Typical normal lymph nodes have been shown to be predominately small and oval, with an echogenic hilum with hypoechoic peripheral cortex, and demonstrate significant hilar vascularity (Image 3).^4^,^5^

Malignant lymph nodes are commonly larger and rounded, with a loss of echogenic hilum; they appear more homogenously hypoechoic and present with peripheral or mixed vascularity with loss of hilar vascularization.^4^,^8^ It is also imperative to apply clinical suspicion when looking at particular locations in the anatomy for lymph nodes compared to abscesses, as lymph nodes are readily identified at the axillary, mediastinal, mesenteric, inguinal, and femoral regions. As compared to abscesses, lymph nodes will not display posterior enhancement, will lack cobblestoning, will not display ultrasonic fluctuance, and will classically appear at expected locations unlike metastases.^4^,^8^ Although we focused on looking at an abscess compared to lymph nodes, ultrasound can be used to identify other anatomic structures as well, including nerves, vessels, and muscle.^1^

## CONCLUSION

Understanding the common sonographic features of abscesses and lymph nodes can allow immediate identification and medical management of the appropriate condition, as evidenced in this case where POCUS imaging changed the medical management of the patient. We suggest that the use of POCUS at bedside can be both a diagnostic tool to provide immediate data to guide distinction of an abscess from a lymph node as well as a tool to educate the patient by sharing and discussing the images at the bedside. Ultimately, this patient’s non-contrast CT imaging was concerning for underlying malignancy. We did not discuss appropriate use of CT imaging to confirm ultrasound findings. The patient was counselled on the findings and instructed that she would likely require a biopsy for confirmation. An incision and drainage was not performed, and the patient was otherwise in stable condition for discharge and had access to short-interval follow-up. Confirmation of the diagnosis is not available as the patient has not followed up with any of our hospital-affiliated clinics per medical records.
